# How is Brazil facing the crisis of Food and Nutrition Security during the COVID-19 pandemic?

**DOI:** 10.1017/S1368980020003973

**Published:** 2020-10-12

**Authors:** Carolina Abreu de Carvalho, Poliana Cristina de Almeida Fonseca Viola, Naiara Sperandio

**Affiliations:** 1 Federal University of Maranhão, Postgraduate Program in Public Health, R. Barão de Itapari, 155-Centro, São Luís, MA 65020-070, Brazil; 2 Federal University of Piauí, Nutrition Department, São Luís, MA, Brazil; 3 Federal University of Rio de Janeiro, Nutrition Course, Av. Aluizio da Silva Gomes, 50-Novo Cavaleiros, Macaé, RJ 27930-560, Brazil

**Keywords:** Food and Nutrition Security, COVID-19, Pandemic, Brazil

## Abstract

The goal of this commentary is to expose the situation of Food and Nutrition Security (FNS) in Brazil in the context of the COVID-19 pandemic by providing a critical analysis of this scenario and suggesting ways to move forward. When COVID-19 arrived in Brazil, a crisis scenario that incorporated economic, social and political aspects became highly visible. This scenario fostered unemployment, poverty and hunger. Besides that, it exposed multiple vulnerabilities that were getting worse over the past few years prior to the pandemic. In this context, COVID-19 found in Brazil a fertile ground for its dissemination and community transmission. The impacts of the suspension of many commercial activities and other economic sectors due to the pandemic were quickly felt socially and economically in Brazil. Some of the actions carried out by the Brazilian government included the emergency aid payment and exemption from payment of energy bills for vulnerable individuals, release of funds for programmes for the direct purchase of food from family farmers, delivery of school food kits directly to students despite the closure of schools and publication of sanitary rules for the operation of restaurants. However, these actions are still insufficient, slow and not sufficiently coordinated to contain the progress of the food and nutritional insecurity crisis in Brazil. The COVID-19 pandemic highlights the urgency for the Brazilian government to again prioritise the FNS agenda. This includes implementing mechanisms to ensure the Human Right to Adequate Food and expanding existing FNS programmes.

## Background

The concept of Food and Nutrition Security (FNS), as adopted in Brazil, was created democratically and with express participation of civil society. It is considered complex, comprehensive and innovative as the dimensions ‘food’ and ‘nutrition’ are combined into a single concept^(1)^.

A human rights approach to FNS, as committed to by Brazil, establishes the responsibility of the country to promote, provide, respect and protect this right. However, by analysing the history of the political agenda of FNS, it is possible to observe a dismantling of the intersectoral policies that were created in order to ensure this right. An expression of this dismantling was the termination of the National Council for Food and Nutrition Security (CONSEA) in January 2019, an important venue for dialogue and articulation between civil societal needs and the governmental priorities^(2)^.

When CONSEA was a section of the National System for Food and Nutrition Security (SISAN), it operated on an agenda that was considered strategic in guaranteeing the FNS led Brazil to be left off of the Hunger Map in 2014^(3)^. The termination of CONSEA jeopardised the decisive agenda that was being consolidated throughout the country and created the possibility of Brazil returning its place on to the Hunger Map^(2)^.

When COVID-19 arrived in Brazil, a crisis scenario that incorporated economic, social and political aspects became highly visible. This scenario fostered unemployment, poverty and hunger. The fight against food insecurity in Brazil was interrupted with the adoption of austerity measures, such as the National Amendment 95/2016^(4)^, which reduced public expenditure on social policies, in addition to labour and social security reforms, which represents a scenario of violation of human rights that are being aggravated by the current pandemic. In this context, the goal of this commentary is to expose the situation of FNS in Brazil in the context of the COVID-19 pandemic by providing a critical analysis of this scenario and suggesting ways to move forward.

## The impact of COVID-19 pandemic in FNS

FAO estimates that the expected number of people in food insecurity by 2020 will increase from 135 million to 265 million due to the COVID-19 pandemic^(5)^. According to the FAO report, the countries most affected by the pandemic are those that have already faced serious food insecurity problems due to conflicts and wars, climate change, and economic crises. In these countries, the risk of food supply chain interruption is imminent, thus damaging farmers’ incomes and the food supply of cities^(5)^.

In Mexico, the country with the third largest number of deaths caused by COVID-19, behind only the United States and Brazil (until August 2020), Vilar-Compte *et al.* ^(6)^ pointed to the absence of interventions and social protective actions by the federal government, especially of those aimed at children. In the United States, the country with the highest number of COVID-19 cases and deaths in the world, trillion-dollar packages have been released to support the country’s economy companies and maintain jobs. Direct income support, increased unemployment benefits and additional support for federal food assistance programmes in the United States have also been implemented^(7)^.

In Brazil, the COVID-19 pandemic exposed multiple vulnerabilities that have been festering over the past few years prior to the pandemic. The latest population surveys carried out in Brazil in the years 2004, 2009 and 2013 indicate that there is regional inequality in the distribution of food insecurity with the North and Northeast regions being the most affected^(8)^. These regions are characterised by a scenario of inadequate access to essential and basic services, which exposes them both to food and nutritional insecurity and to the spread of COVID-19. This inadequacy is demonstrated by the fact that these were also the regions most affected at the beginning of the pandemic. This finding shows that the social determination of food insecurity in Brazil is similar to that of the COVID-19 pandemic, affecting mainly people in racial, social, economic and sanitary vulnerability^(9)^. In this context, COVID-19 found fertile ground in Brazil for its dissemination and community transmission.

The impact of the suspension of many commercial activities and other economic sectors due to the pandemic was quickly felt socially and economically in Brazil. The comparison of the final quarter of 2019 and first quarter of 2020 revealed an increase in the unemployment rate, which was the highest in the Northeastern region and caused a higher number of poor and extremely poor people in Brazil than was already there^(10)^. By June 2020, more than 10 million workers had already had their job contracts suspended or had their working hours and salaries reduced, and the country had more than 1·2 million people unemployed.

The increase in unemployment rates and poverty in the country in addition to the increase in prices of natural or minimally processed food during the pandemic^(11)^ indicate the addition of millions of Brazilians to the group of people who are vulnerable to food and nutrition insecurity. It is important to highlight that at the beginning of the pandemic, the population was concerned with the lack of food, but currently, no signs of food shortage in Brazil are visible. Therefore, although no interruption in food supply occurred, Brazilians with compromised incomes during the pandemic may have problems accessing food as they are unable to afford it.

A study conducted in Brazil by the UNICEF and the Brazilian Institute of Public Opinion and Statistics (IBOPE) from 3 July 2020 to 18 July 2020, showed that during the pandemic, one in five Brazilians aged 18 years or older (33 million) experienced an episode of having no money to buy food when their income ran out. This study also reports that about nine million Brazilians were unable to have a meal because there was no food or money to buy one^(12)^.

## Government responses

Given the evident and expected impacts caused by the COVID-19 pandemic in relation to the population’s FNS, the Brazilian government responded with a few actions although they were slow and uncoordinated. Furthermore, it is important to stress that the majority of these actions were pressured and amplified by the National Congress. The main governmental action to minimise the impacts of the pandemic over the income of the most vulnerable families was the introduction of an emergency aid payment in the amount of US$120 over a 5-month period for informal workers, individual entrepreneurs, independent workers and the unemployed. The initial proposal of the federal government was only US$40, but the amount was increased after Congressional intervention. Up to 3 August 2020, 66·2 million Brazilians had already received this payment. However, the way this aid has been operationalised has been heavily criticised due to the difficulties in access to registration for some population groups and delays in processing registrations^(11)^.

Moreover, an announcement about the expansion of the income transfer programme Bolsa Família, aiming to release more than US$680·000·000 has been made. However, until 18 August 2020, only 12·2 % of the amount allocated to the expansion had been used^(13)^. Another measure implemented to preserve family incomes was payment exemption of up to 220 kWh/month towards the electricity bill of low-income families from 1 April to 30 August.

The Program for Food Acquisition (PAA) is one of the main FNS programmes in Brazil. This programme’s main goal is to promote access to food and encourage family agriculture. Due to COVID-19, this programme received a budget supplementation of 500 million reais, which is considered insufficient^(14)^, especially considering the scenario caused by the pandemic, and delayed since the publication of the provisional measures only happened almost 2 months after COVID-19 hit Brazil^(11)^. This emergency act took place after recent stripping of resources allocated to PAA, which were reduced from US$117·200·000 in 2012 to US$12·600·000 in 2019^(14)^.

The National School Nutrition Program (PNAE)^(3)^ is the oldest public policy for FNS in Brazil. With schools closing as a strategy to reduce the spread of the virus, millions of students have become vulnerable to food and nutrition insecurity. As a way to mitigate the effects of the pandemic on the FNS of this part of the population, the federal government authorised the use of school nutrition resources for the distribution of food to students. In this programme, 30 % of the purchased food should come from family agriculture. Therefore, purchasing of this food even during the pandemic allows the continuity of the outflow of production and farmers’ income.

With respect to the sanitary and hygienic aspects of FNS, the food supply chain and the food industry also needed to adapt to changes in food safety protocols imposed by the pandemic. Although there is little evidence of transmission of COVID-19 through food, it is important to highlight that there are potential sources of risk of contamination along the food supply chain that involve many people, surfaces and environments. In this sense, to ensure food and environmental safety, strict safety protocols are necessary^(15)^.

The National Health Surveillance Agency (ANVISA) has published sanitary guidelines in relation to COVID-19 and the Good Practices for Manufacturing and Manipulation of Food^(16)^. However, we believe that the capacity of food safety inspectors is limited when it comes to implementing the recommendations in this publication after considering that before the pandemic ANVISA already had difficulties in satisfying all of its inspection demands.

## Suggestions to improve the responses for the Food and Nutrition Security crisis

The impact of the pandemic on the FNS of the Brazilian population was strengthened as the country faced a previous scenario of progressive weakening of FNS policies marked by the termination of CONSEA, budget cuts for programmes for promotion and support of family agriculture, and the disbanding of these programmes. These policy and institutional changes undermine the capability of the State to satisfy the demands that the COVID-19 pandemic has imposed on the FNS of the population. However, in the current scenario, we believe that a few actions would result in better responses to FNS protection. Among those, we highlight the urgent need for actions imposed by the State and then coordinated among the three levels of the government. The need for coordinated and intersectoral actions to address FNS is even more important during a pandemic. Another recommendation is to harness the great potential of Primary Healthcare to implement FNS programmes in territories. Primary health care has been underused when it comes to identification and attention to the needs of families regarding FNS.

Mobility restrictions recommended as a way to prevent the dissemination of COVID-19 and the work-from-home conditions imposed on thousands of Brazilians in addition to full-time childcare could make families seek more practical and quicker foods, such as ultra-processed and fast food items. In the context of the COVID-19 pandemic, it is possible that the population will further increase the intake of ultra-processed foods and aggravate the situation of food insecurity thus leading to worsening of the dietary quality^(12)^. Evidence that the diet has worsened during the pandemic in Brazil can be found as there was a decrease in the consumption of fruits and vegetables and an increase in the consumption of several types of ultra-processed foods. Together with the deterioration of dietary quality, increased sedentary behaviour demonstrated by longer screen time and a drastic decrease in physical activity practice has been reported in Brazil during the pandemic^(12,^ ^17)^.

The perpetuation of these inadequate food and lifestyle habits during the months of quarantine has resulted in an increase in the prevalence of overweight and other chronic noncommunicable diseases, such as hypertension, cardiovascular diseases and diabetes. Therefore, actions to promote healthy eating and physical activity in the context of social isolation must be implemented.

The weakening and the attempt to disarticulate social control, especially represented by the termination of CONSEA, is also an aspect that limits and hinders the capability to respond to actions for improving FNS during the pandemic. Nevertheless, some of the actions that have taken place to date happened under great pressure from civil society since a remnant of organised structure of social control still exists.

Finally, with the impacts of the COVID-19 pandemic, another action which has become even more urgent is that the government again should prioritise the FNS agenda and update the National Plan for Food and Nutrition Security (PNSAN). The last one ended in 2019, and the replacement that was supposed to be implemented from 2020 to 2023 has not been created. Updating the PNSAN would allow for the creation of coordinated actions with cities and municipalities and budget transfers that are aligned with the needs of the programmes and public policies. This would ensure again the realisation of the Human Right to Adequate Food and expand FNS programmes.

Strengthening human rights, including FNS, is essential for the structuring of a more equitable society, and it has a legal basis through the Constitution. However, it is a historical challenge that will become even more complex in the context of a pandemic and runs counter to political will and commitment of leaders and nations.
